# A Study of the Degree of Gall Bladder Wall Thickness and Its Impact on Patients Undergoing Laparoscopic Cholecystectomy

**DOI:** 10.7759/cureus.38990

**Published:** 2023-05-14

**Authors:** Irshad Khan, Parul Yadav, Rama K Saran, Sarthak Sharma, Amit K Sharma

**Affiliations:** 1 General Surgery, Dr. Sampurnanand Medical College, Jodhpur, IND

**Keywords:** cholecystitis, laparoscopic cholecystectomy, ultrasonography, gall bladder thickness, gall stone disease

## Abstract

Background

The gold standard management for symptomatic gallstone disease is elective laparoscopic cholecystectomy, which has replaced open cholecystectomy. The wall thickness of the gallbladder is an indicator of cholecystitis in patients who have presented with symptoms of gallstone disease. The aim of this study was to evaluate preoperative gall bladder wall thickness by ultrasonography and assess its impact on the outcome of laparoscopic cholecystectomy, including conversion rate, complications, operative time, and postoperative hospital stay.

Method

This prospective study was conducted on 350 patients with symptomatic gallstone disease, those who had undergone laparoscopic cholecystectomy in Dr. Sampurnanand Medical College, Jodhpur, and attached hospitals from July 2019 to November 2021. On the basis of ultrasonography findings of gallbladder wall thickness, patients were divided into four groups: normal thickness - up to 2 mm, mild thickness - 3-4mm, moderate thickness - 5-6mm, and severe wall thickness - more than 6mm). Up to 2 millimeters thickness was considered as normal.

Results

The incidence of conversion rate, as well as intra or postoperative complications, were higher in moderate and severe wall thickness groups. The maximum incidence of complication rate is seen in moderately thickened group (33.33%). In severely thickened group, complication was seen in 100% of patients. Operative time, as well as postoperative hospital stay, were more in higher thickness groups. There was a statistically significant correlation between gallbladder wall thickness and conversion rate, complications operative time, and postoperative length of stay.

Conclusion

Increased gallbladder wall thickness causes increased intra as well as postoperative complications, more conversion to open procedure rate, increased operative time, and enhanced postoperative hospital stay. Among the total study population, 29.71% of patients had increased gallbladder wall thickness. In our study, a positive correlation was seen among gallbladder wall thickness, complication rate, conversion rate, intraoperative time, and postoperative hospital stay.

## Introduction

The gold standard management for symptomatic gallstone disease is elective laparoscopic cholecystectomy, which has replaced open cholecystectomy [1]. The wall thickness of gallbladder is an indicator of cholecystitis in patients who have presented with symptoms of gallstone disease [2]. The gallbladder wall is normally measured less than 3mm by ultrasonography (USG) [3]. Gallbladder wall thickness measurement is accurate to within 1 mm in 93% of patients by USG [4]. A thickness of the gallbladder wall of more than 3 mm is suggestive of cholecystitis [5-7]. Despite the fact that this is also found in many conditions like gallbladder cancer and adenomyomatosis, it is the hallmark of cholecystitis, but in these conditions, the diameter of the gallbladder is directly part of the underlying pathology [8-9]. For laparoscopic cholecystectomy, the prediction of conversion and complications are quite important for the management of gallstone disease [10]. Gallbladder wall thickness is one of the major factors in determining the type of surgical procedure for the management of gallstone disease. The aim of this study was to evaluate the impact of gallbladder wall thickness on the outcome of laparoscopic cholecystectomy for gallstone disease and to establish the fact that gallbladder wall thickness is an important preoperative marker for prediction of intra and postoperative complications, conversion to open procedure, intraoperative time and, postoperative length of stay.

## Materials and methods

This prospective observational study was conducted at Mathura Das Mathur Hospital and Mahatma Gandhi Hospital attached to Dr. Sampurnanand Medical College (SNMC), Jodhpur, Rajasthan, India, from 1 July 2019 to November 2021. This study comprises of a total of 350 cases. This study started after taking permission from the College Institutional Ethical Committee (IEC) (SNMC/IEC/2019/1383-1385 ). Dr. SNMC IEC have access to any information or data of this study.

All diagnosed cases of symptomatic gallbladder disease (calculous cholecystitis, cholelithiasis, and other surgical inflammatory gallbladder pathology) planned for laparoscopic cholecystectomy were included in this study. The exclusion criteria included deranged coagulation profile (prothrombin time), acute biliary pancreatitis, carcinoma gallbladder, all types of gallbladder polyps, cholangitis, perforated gallbladder, biliary enteric fistula, portal hypertension, peritonitis, and pregnancy. 

The data was collected as general details of patients, including their name, age, sex, registration number, date of admission, date of operation, and date of discharge. A detailed history of the patient with special emphasis on symptoms of biliary colic, history of previous attacks of colic, jaundice, history suggestive of pancreatitis, etc. History of comorbid conditions, including diabetes mellitus (DM), hypertension (HT), significant illnesses and previous symptoms, general physical examination, and abdominal examination. All patients presented with symptoms suggestive of uncomplicated or complicated gallstone disease - having pain in the epigastrium or right hypochondrium and increased severity after taking food, flatulence, and fever. All patients were thoroughly examined and further evaluated with abdominal ultrasonography by a sonologist. The gallbladder wall thickness was measured at the level of the fundus of the gallbladder along with other parameters of gallstone disease. On the basis of ultrasonography findings of gallbladder wall thickness, patients were divided into four groups: normal thickness - up to 2 mm, mild thickness - 3-4mm, moderate thickness - 5-6mm, and severe wall thickness - more than 6mm). Up to 2 millimeters thickness was considered as normal.

Patients who were diagnosed with gallstone disease were advised to undergo laparoscopic cholecystectomy. Those willing to undergo surgery were then divided into four groups on the basis of gallbladder wall thickness. Patients were then planned for laparoscopic cholecystectomy either during the same sitting or different according to patient presentation and preference. Their intraoperative findings, duration of surgery, rate of conversion to open procedure, and length of hospital stay were recorded. Intraoperative and postoperative complications were recorded, and they have been broadly categorized as intraoperative and postoperative complications as adhesions, hemorrhage, common bile duct (CBD) injury, postoperative bile leak, and surgical site infection. Patients were followed up till suture removal or till postoperative day 12. Late complications were not included.

## Results

In our study, the youngest patient was 18 years of age, and the oldest was 90 years of age. The majority of the patients, 92 (26.29%), were present in the age group of 41-50 years. Forty-one (11.71%) patients were >60 years of age. Maximum complications were present in seven (17.07%) in the >60 age group. The mean age of presentation was 45.01 years. The mean age of males was 46.64 years with a standard deviation of 12.64 years, and the mean age of females was 45.01 years with a standard deviation of 13.97 years.

In our study, of the patients who underwent laparoscopic cholecystectomy, 90 (25.71%) were male, and 260 (74.29 %) were female, with a male-to-female ratio of 1:2.8. Complications were present in nine (10%) male and in 12 (4.61%) of female patients.

The incidence of conversion seen in the moderately thickened group was 40%. The conversion rate was 100% in the severely thickened group, whereas it was 6.67% in the normal gall bladder wall thickness group. The correlation between gallbladder wall thickness and conversion rate is statistically significant (p=value <0.0001), and the association is positive.

Table 1 shows the correlation between gallbladder wall thickness and the incidence of conversion to open procedure.

**Table 1:** Correlation between gallbladder wall thickness and incidence of conversion to open procedure

Gallbladder thickness	Total no. of patients	Conversion			
		Yes		No	
		N	%	N	%
Normal	246	1	6.67	245	73.13
Mild	84	3	20.00	81	24.18
Moderate	15	6	40.00	9	2.69
Severe	5	5	33.33	0	0.00
Total	350	15	100.00	335	100.00

**Figure 1:**
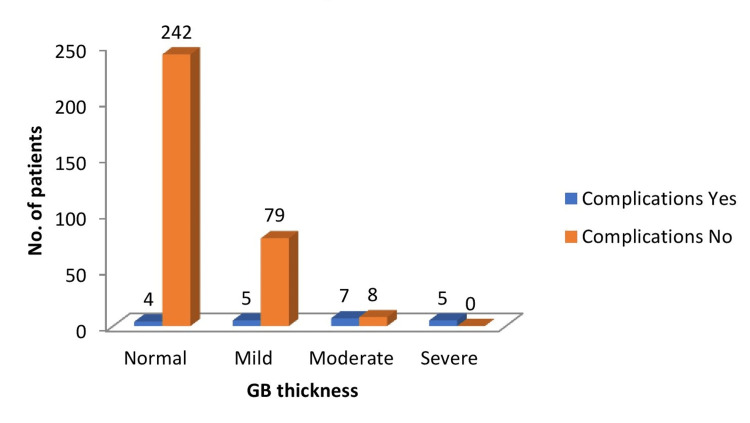
Correlation between gallbladder wall thickness and incidence of complication rate GB - gallbladder

Complications were seen in a total of 21 patients out of 350 patients (6%), either intra or postoperatively. The incidence of complication rates was 100% in the severely thickened group, whereas it was 33.33% in the moderately thickened group. In the severely thickened group, complications were seen in all patients. The correlation between gallbladder wall thickness and intra and postoperative complications is statistically significant (p-value <0.0001) (Figure 1).

A longer mean operative time was seen in the severely thickened group (126.4 minutes), followed by the moderately thickened group (89 minutes), and the shortest operative time was seen in the normal group (52.97 minutes). The correlation between gallbladder wall thickness and mean operative time is statistically significant.

Maximum postoperative hospital stay was seen in the severely thickened group (11 days, standard deviation 10.12 days), followed by the moderately thickened group (4.8 days), and the shortest mean postoperative hospital stay was seen in the normal gallbladder wall thickness group (2.8 days, standard deviation 1.45 days). The correlation between gallbladder wall thickness and mean postoperative hospital stay is statistically significant (p-value <0.0001).

Table 2 shows the correlation of gallbladder wall thickness with operative time and postoperative hospital stay, and Table 3 shows intra and postoperative complications in each group.

**Table 2:** Correlation of gallbladder wall thickness with operative time and postoperative hospital stay

Gallbladder thickness	Operative time		Postoperative hospital stay	
	Mean	SD	Mean	SD
Normal	52.97	9.25	2.8	1.45
Mild	60.59	14.99	3.02	1.57
Moderate	89	31.82	4.8	2.11
Severe	126.4	2.6	11	10.12
p-value	<0.0001		<0.0001	

**Table 3:** Intra and postoperative complications in each group ERCP - endoscopic retrograde cholangiopancreatography

Complications	Gallbladder thickness				Total
	Normal	Mild	Moderate	Severe	
Normal	242	79	8	0	329
Bile leak	1	1	1	2	5
Hemorrhage	0	1	4	2	7
Jaundice PO ERCP	1	1	0	0	2
Wound site infection	2	2	2	1	7
Total	246	84	15	5	350

## Discussion

Currently, laparoscopic cholecystectomy is the gold-standard surgical procedure for gallstone disease. Although a minority of them still convert to open procedures due to impaired visualization of surgical anatomy due to inflammation, dense adhesions, abnormal anatomy, or any other region. Few of them have intra or postoperative complications. Identification of predictive factors of conversion and complications can be beneficial to both surgeon as well as for patient outcomes. 

As far as demographics were concerned, the male-to-female ratio (M:F) in our study was 1:2.8, which is similar to an Indian study by Chandra et al. in which the sex ratio was 1:1.64 [11]. This is in contrast to Western studies [12].

Many preoperative factors have been identified in the literature for the prediction of impact on laparoscopic cholecystectomy, but gallbladder wall thickness is a very important factor for laparoscopic cholecystectomy. The cutoff value for gallbladder wall thickness is 2 millimeters which is measured by ultrasonography by an expert radiologist.

As per the results of the present study, the thicker the gallbladder wall, the greater the risk of conversion to open procedure and increased risk of intra as well as postoperative complications. Similarly, it increases operative time as well as the total duration of hospital stay.

The overall conversion rate to open procedure was 4.28%. It is slightly less than the reported literature; 5-10% [13] and similar to a study done by Chandra et al. [11]. The conversion rate was higher in the thicker gallbladder wall group and it had a positive association which is similar to the literature. In the majority of cases, the cause for conversion was dense inflammatory adhesions, which were secondary to cholecystitis or empyema gallbladder. These adhesions made safe dissection impossible and led to conversion.

The overall complication rate in the present study was 6%, which included both intra and postoperative complications. Postoperative complications were more prevalent than intraoperative complications. The incidence of postoperative complications was 4%, which is slightly less than the available literature [8]. The most common postoperative complication was bile leak which was stopped spontaneously after the 10th postoperative day; only four patients needed endoscopic retrograde cholangiopancreatography (ERCP)-guided stenting.

Operative time and postoperative length of stay were more criteria that were included in the present study. The lowest mean operative time was seen in the normal wall thickness group; 52.97 minutes with SD of 9.27 minutes. The mean operative time increased with increasing gallbladder wall thickness; this is similar to the literature [14].

Another criterion that was included in this study was the postoperative length of stay as a measurement of outcome after surgery. The mean postoperative time was the lowest in the normal gallbladder wall thickness group, 2.8 days with SD of 1.45 days, and the highest in severely thickened group, 11 days with SD of 10.12 days. The contributory factors for increasing length of stay were postoperative bile leak, postoperative pain, and surgical site infection. The postoperative length of stay is slightly higher than the reported literature [14].

There were a few limitations of this study; one of them was the timing of ultrasonography (USG). In some patients, USG was done in an elective setting, and in some, it was done in an acute setting in an emergency when the patient presented with acute abdominal pain in case of cholecystitis. Ultrasonography itself is a highly subjective investigation, although it was done by a single expert radiologist.

There can be discrepancies in acute settings about the line of management; it could be prudent to wait for surgery in case of acute cholecystitis to avoid complications, but no proven study is yet available for this; it depends on the operating surgeon's experience.

## Conclusions

Increased gallbladder wall thickness increases intra as well as postoperative complications, more conversion to open procedure rate, and increases operative time as well as the postoperative hospital stay. Among the total study population, 29.71% of patients had increased gallbladder wall thickness. In our study, a positive correlation was seen among gallbladder wall thickness, complication rate, conversion rate, intraoperative time, and postoperative hospital stay. An accurate preoperative or intraoperative diagnostic tool is mandatory for planned gallbladder surgery to provide information and to predict the outcome of laparoscopic cholecystectomy.

Ultrasonography is a highly subjective imaging modality, but with the availability of an expert radiologist, an assessment of gallbladder wall thickness was done at an accurate level. More than 2 mm gallbladder wall thickness was considered significant, and all findings were significantly high in these groups. It appears that gallbladder wall thickening is the best predictor for outcome following laparoscopic cholecystectomy.
